# Supplementary material for: RAGE as a Novel Biomarker for Prostate Cancer: A Systematic Review and Meta-Analysis
Source: Cancers (Basel). 2023 Oct 9;15(19):4889. doi: 10.3390/cancers15194889 (PMC10571903; doi:10.3390/cancers15194889)
Supplement: Supplementary file 1 [file cancers-15-04889-s001.zip › Supplementary Table S1_RAGEinPCa_QAtable.pdf]

Supplemental Table S1. Quality assessment of included clinical studies.

Source	Selection				Comparability[5]	Exposure			Total[9]
Author, Year	Definition[1]	Representative[2]	Selection[3]	Definition[4]		Ascertainment[6]	Method[7]	Technique[8]	
**Aboushousha, 2019 [36]**	★	★	★	0	0	★	0	★	5
**Akkus, 2020 [37]**	★	★	★	0	★	★	★	★	7
**Foster, 2014 [38]**	★	0	0	0	0	★	0	★	3
**Hemani, 2005 [39]**	★	★	★	★	0	★	★	★	7
**Ishiguro, 2005 [40]**	★	★	★	★	★	★	★	★	8
**Konopka, 2020 [24]**	★	★	0	0	★	★	★	★	6
**Kuniyasu, 2003 [41]**	★	★	0	0	★	★	0	★	5
**Ravenna, 2009 [42]**	★	★	★	0	0	★	0	★	5
**Zhao, 2014 [43]**	★	★	★	0	0	★	★	★	6

[1]Indicates that cases are independently validated for prostate cancer (PCa) by a trained histopathologist (0, 1 star); [2]cases are from a representative population or drawn from the same community (0, 1); [3]control of non-PCa prostate tissue (prostatitis, benign prostate hyperplasia, or healthy prostate) was included (0, 1); [4]control of normal prostate was included (0, 1); [5]specified as non-metastatic vs. metastatic or androgen-sensitive vs. androgen-insensitive PCa (0, 1, 2); [6]PCa samples were attained from medical records (0, 1); [7]collection method of PCa tissue was included (0, 1); [8]approved identification technique was used to measure RAGE expression (0, 1); [9]total: minimum equals 1 star; maximum equals 9 stars.
