# Supplementary material for: RAGE as a Novel Biomarker for Prostate Cancer: A Systematic Review and Meta-Analysis
Source: Cancers (Basel). 2023 Oct 9;15(19):4889. doi: 10.3390/cancers15194889 (PMC10571903; doi:10.3390/cancers15194889)
Supplement: Supplementary file 1 [file cancers-15-04889-s001.zip › Supplementary Table S2_RAGEmeta_InVitroQA.pdf]

Supplementary Table S2. Quality assessment of included cell culture studies (adapted from WCRF/UoB recommendations) [28].

Author, Year	Source[1]	Experimental Conditions					Selective Reporting[7]	Statistics[8]	Total
		Culture Conditions[2]	Replicates[3]	Positive Controls[4]	Negative Controls[5]	Multiple Cell Lines[6]			
**Bao et al., 2015 [44]**	1	1	1	1	1	0	1	1	7
**Elangovan et al., 2011 [45]**	1	1	1	1	1	1	1	1	8
**Ishiguro et al., 2005 [40]**	1	1	1	0	1	0	1	1	6
**Siddique et al., 2013 [46]**	1	1	1	1	1	0	1	1	7
**Wu et al., 2021 [47]**	1	1	1	0	1	1	1	1	7
**Zhang et al., 2018 [21]**	1	1	1	1	1	0	0	0	5

Studies are given a score of 0 or 1 for each of the following parameters (a score of 0 was given for a lack of criteria fulfillment or failure to report): [1]cell lines are independently validated; [2]comparable culture conditions to other studies; [3]experiment performed in replicate(s); [4]appropriate positive controls included; [5]appropriate negative controls included; [6]more than one cell line used; [7]all experimental results are reported (a score of 0 was given for missing statistical data); [8]appropriate statistical test used.
