# Supplementary figures and images for: RAGE as a Novel Biomarker for Prostate Cancer: A Systematic Review and Meta-Analysis
Source: Cancers (Basel). 2023 Oct 9;15(19):4889. doi: 10.3390/cancers15194889 (PMC10571903; doi:10.3390/cancers15194889)

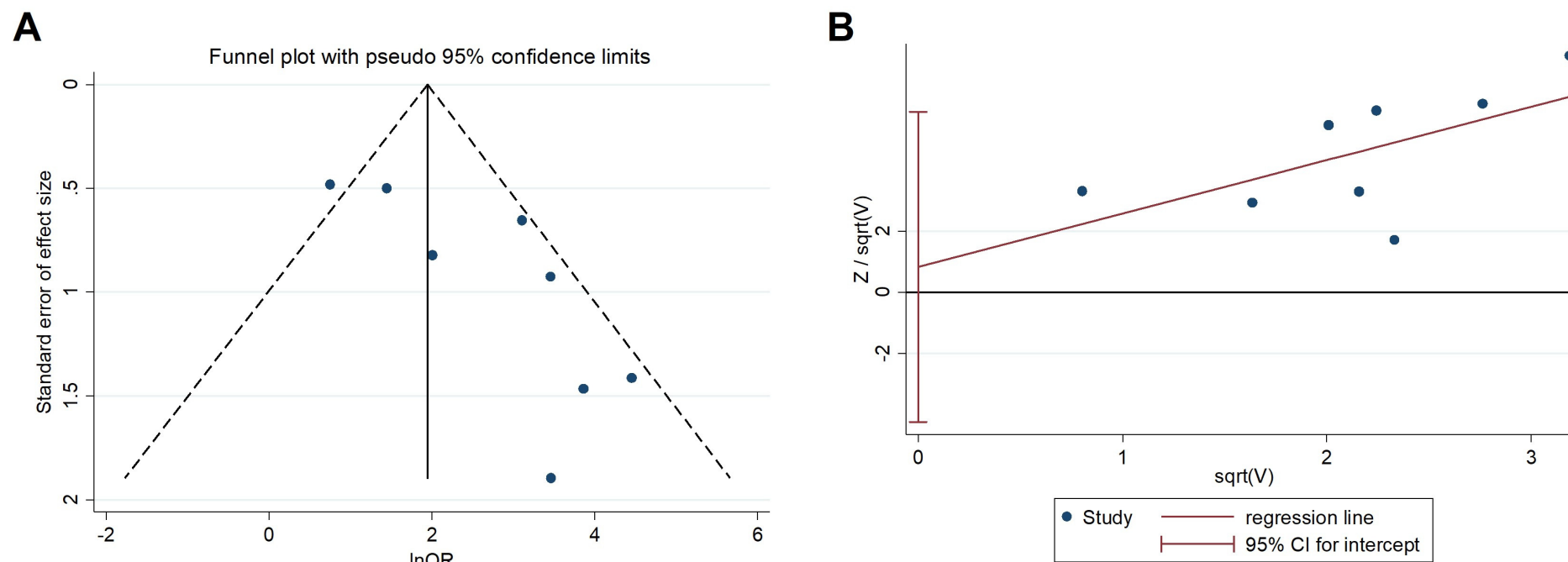

**Figure S1:** (A,B) Results of tests of bias.

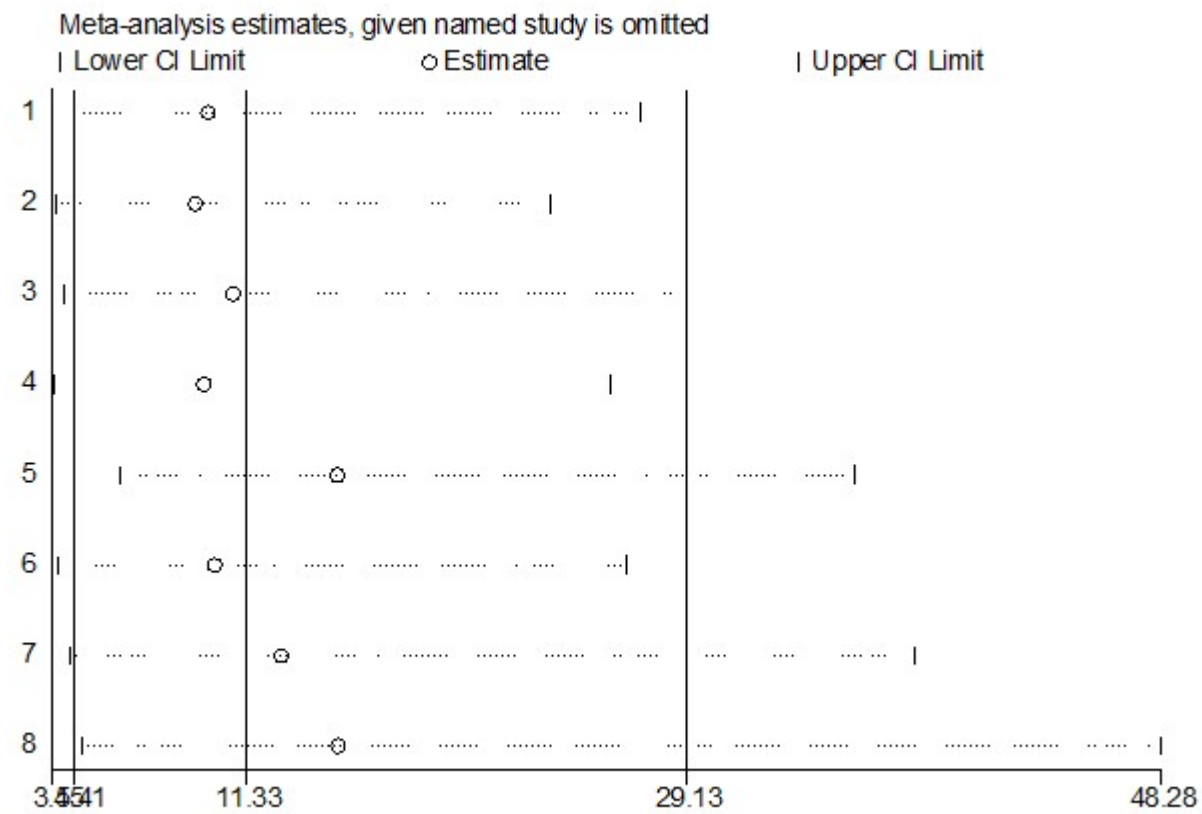

**Figure S2:** Leave-one-out meta-analysis.

Supplement: Supplementary file 1 [file cancers-15-04889-s001.zip › Supplementary Figures S1 and S2.pdf]
